# Primary temporal bone osteosarcoma presenting as a parotid tumor: A case report with diagnostic challenges

**DOI:** 10.1016/j.radcr.2025.07.012

**Published:** 2025-08-05

**Authors:** Naho Okada, Kana Miyatake, Hitomi Iwasa, Miki Nishimori, Kosuke Nakaji, Noriko Nitta, Rika Yoshimatsu, Tomoaki Yamanishi, Tomohiro Matsumoto, Hiroaki Ito, Mitsuko Iguchi, Takuji Yamagami

**Affiliations:** a Department of Diagnostic and Interventional Radiology, Kochi Medical School, Kochi University, Kochi, Japan; b Department of Otolarygology, Head and Neck Surgery, Kochi Medical School, Kochi University, Kochi, Japan; c Department of Diagnostic Pathology, Kochi Medical School, Kochi University, Kochi, Japan

**Keywords:** Temporal bone, Parotid gland, Osteosarcoma

## Abstract

Osteosarcoma rarely arises from the temporal bone, and few primary cases have been reported in adults. The prognosis of head and neck osteosarcoma is generally poor because of the complex anatomy in the region. Complete resection with negative margins is essential to adequately treat head and neck osteosarcoma. Preoperative imaging is crucial given the importance of complete surgical resection to prognosis. In this case, preoperative imaging, including computed tomography and magnetic resonance imaging, revealed a lobulated mass with coarse internal calcification, displacing the parotid gland and extending into the parapharyngeal space. The lesion lacked abnormal findings in the adjacent temporal bone on imaging, but a sarcomatous mass with central calcification could be the basis for including osteosarcoma in the differential diagnosis. We report this case of primary osteosarcoma originating from the temporal bone that was initially discovered as a parotid tumor.

## Introduction

Osteosarcoma rarely originates from the temporal bone [1], with few primary cases described in adults. The prognosis of head and neck osteosarcoma is generally worse than that of osteosarcoma of the extremities [2], and preoperative imaging is highly significant for complete surgical resection. In this study, we report a case of primary osteosarcoma of the temporal bone that was discovered as a parotid tumor together with a literature review.

## Case presentation

A 52-year-old woman visited her previous doctor because of swelling and pain in the right parotid gland. Her symptoms were alleviated with oral medication, but her pain reappeared after 1.5 months. She was referred to our hospital because of sensory loss at the right corner of her mouth.

She underwent surgery for breast cancer (ductal carcinoma in situ) 2 years before presentation, and she was receiving hormone therapy at the time of presentation. She had no history of radiation exposure or trauma. The clinical examination revealed a fixed mass measuring 5 × 4 cm2 in the right parotid gland. Blood testing revealed ALP elevation (645 U/L). No other abnormalities were identified by biochemical or blood testing.

**Fig. 1:**
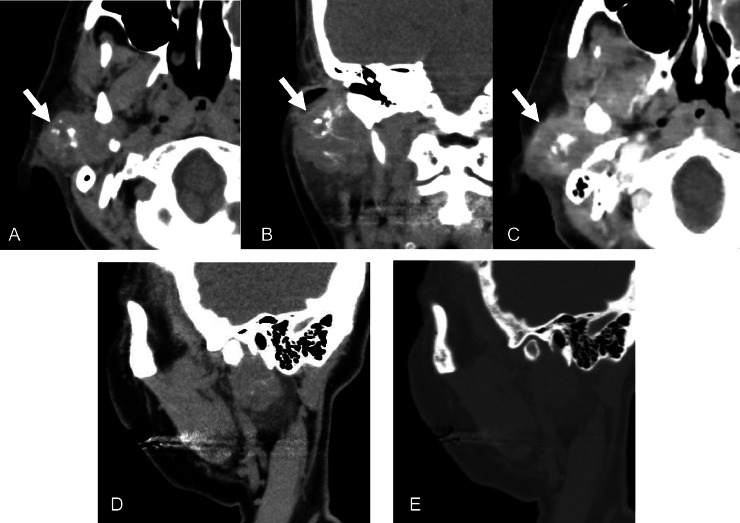
(A and B) Axial and coronal computed tomography revealed a lobulated mass that displaced the right parotid gland and extended into the parapharyngeal space. The boundary of the mass was partially obscured. The lesion exhibited coarse internal calcification. (C) On axial enhanced computed tomography, the mass exhibited heterogeneous contrast enhancement with marginal predominance. (D and E) Sagittal computed tomography revealed no apparent destruction or infiltration of the surrounding bone tissue.

**Fig. 2:**
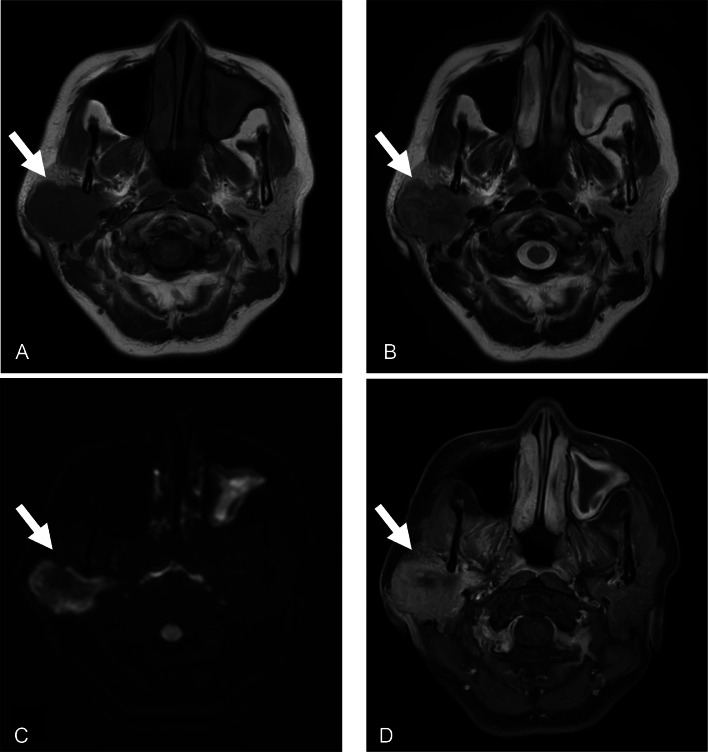
(A) Axial T1-weighted imaging revealed low signal intensity. (B) Axial T2-weighted imaging revealed heterogeneous low signal intensity. (C) Axial diffusion-weighted imaging revealed high signal intensity. (D) Contrast-enhanced T1-weighted imaging revealed a contrast effect with limbal predominance and a contrast-deficient area suspicious for internal necrosis.

**Fig. 3:**
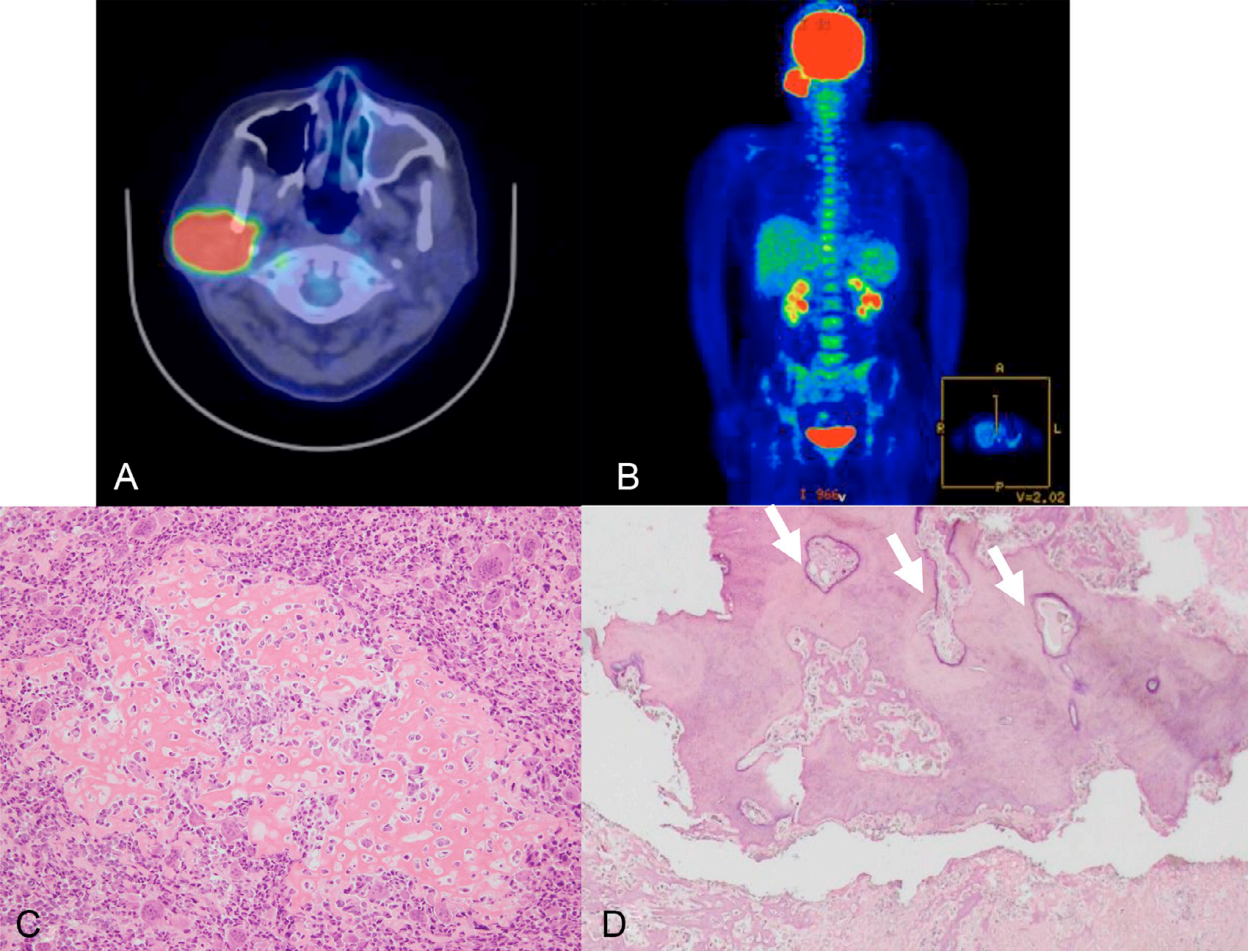
(A and B) 18F-fluorodeoxyglucose positron emission tomography/computed tomography revealed a maximum standardized uptake value of 18.9 in the mass and no abnormal uptake in other areas. (C) Histopathological examination by hematoxylin and eosin staining revealed the dense proliferation of spindle to polygonal-shaped cells of varying size and prominent nuclear staining, lacking apparent cohesive features, as well as abundant interspersed osteoclast-like multinucleated giant cells. Lace-like osteoid and well-developed bone matrix were widely observed, accompanied by calcification. (D) The tumor invades the marrow cavity of the temporal bone (arrow).

Enhanced computed tomography (CT) of the head disclosed a 35-mm-long lobulated mass that displaced the right parotid gland and extended into the parapharyngeal space (Fig. 1A and B). The boundary of the mass was partially obscured. The lesion exhibited coarse internal calcification and heterogeneous contrast enhancement with marginal predominance (Fig. 1A, B and C). The lesion did not apparently destroy or infiltrate the surrounding bone (Fig. 1D and E). Low signal intensity was noted on T1-weighted imaging (Fig. 2A), heterogeneous low signal intensity was observed on T2-weighted imaging (Fig. 2B), high signal intensity was detected on diffusion-weighted imaging (Fig. 2C), and a decreased apparent diffusion coefficient was recorded. Contrast-enhanced T1-weighted imaging uncovered a contrast effect with limbal predominance and a contrast-deficient area suspicious for internal necrosis (Fig. 2D). On 18F-fluorodeoxyglucose positron emission tomography/CT, the lesion exhibited a maximum standardized uptake value of 18.9. No abnormal uptake was present elsewhere (Fig. 3A and B). Fine-needle aspiration cytology was performed on suspicion of salivary duct carcinoma or carcinoma ex pleomorphic adenoma. Extended right parotidectomy was performed because of the suspicion of malignancy.

The pathological examination revealed a 5-cm irregular white tumor on the cut surface within a 13-cm specimen, which included part of the auricle and temporal bone. Histologically, there was dense proliferation of spindle-to-polygonal–shaped cells with prominent nuclear staining and varying sizes, albeit lacking cohesive features, along with abundant interspersed osteoclast-like multinucleated giant cells. Lace-like osteoid and well-developed bone matrix accompanied by calcification were widely observed (Fig. 3C). The tumor extended superiorly to involve the parotid gland and displayed continuity with the marrow cavity of the temporal bone at some points (Fig. 3D). Based on the histopathological features, a diagnosis of osteoblastic osteosarcoma of the temporal bone was rendered.

Because the surgical margin was positive, the patient underwent 3 cycles of adjuvant chemotherapy with cisplatin and doxorubicin. Two years later, surveillance computed tomography revealed a 10-mm nodule in the right lung. The lesion was excised via video-assisted thoracoscopic wedge resection, and histopathology confirmed pulmonary metastasis of osteosarcoma. The patient has remained under imaging surveillance, and at the 4-year follow-up, no local recurrence or additional metastatic disease has been detected.

## Discussion

Osteosarcoma is a malignant tumor that mostly arises in the long bones of the extremities, with head and neck involvement occurring in fewer than 10% of all cases. Compared with osteosarcoma of the extremities, head and neck osteosarcoma tends to occur in the third or fourth decades of life [2]. In middle aged and older adults, osteosarcomas often occur secondary to Paget’s disease of bone or exposure to radiation or chemotherapeutics [3]. Head and neck osteosarcoma typically occurs in the maxilla, zygoma, and mandible, with few reports describing tumors in the temporal bone. To our knowledge, approximately 11 cases of temporal bone osteosarcoma in the pediatric population have been reported in the English literature, including both primary and secondary cases [4]. In adults, fewer than 10 cases of primary temporal bone osteosarcoma have been reported in the English literature [[5], [6], [7], [8], [9], [10]].

Craniofacial osteosarcomas are osteolytic on plain X-ray in 2-thirds of patients, and they are typically characterized by a long transition zone. Periosteal reactions are rarely observed, and calcifications are present in more than half of all cases [11]. CT uncovers osteolytic, mixed osteolytic/osteoblastic, and osteoblastic changes in 40%, 35%, and 19% of cases, respectively. An irregular or punctate osteoid matrix was observed in both the extraosseous soft tissue component and the osseous lytic region in 91.6% of cases. Luo et al. investigated the imaging features of osteosarcomas arising in irregular and flat bones, and CT demonstrated various patterns of bone destruction in all 83 cases [12]. However, in the present case, no distinctive lesions were observed in the adjacent cranial bone on preoperative imaging. In such cases, diagnosis based on preoperative imaging can be difficult.

The notable imaging findings in the present case included coarse calcification within the tumor. Although parotid tumors with calcification are rare, the differential diagnoses include salivary duct carcinoma, carcinoma ex pleomorphic adenoma, mucoepidermoid carcinoma, pleomorphic adenoma, and metastatic tumors. Salivary duct carcinoma is a high-grade adenocarcinoma arising from the ductal epithelium, and 33%–50% of patients exhibit calcification on CT [13]. Carcinoma ex pleomorphic adenoma arises from benign pleomorphic adenoma, with 39.6% of patients displaying calcification on CT [[14], [15]].

Conversely, extraparotid lesions suggest the possibility of extraskeletal osteosarcoma. Extraskeletal osteosarcoma is an osteogenic soft tissue tumor that lacks attachment to skeletal tissue, and it is often associated with prior radiation exposure or trauma. Similarly as other soft tissue sarcomas, extraskeletal osteosarcoma predominantly occurs in middle-aged and older adults. The imaging findings of extraskeletal osteosarcoma are mostly nonspecific. Typically, plain X-ray appears normal, T1-weighted imaging reveals heterogeneous signals ranging from iso- to hyperintense relative to those in skeletal muscle, and T2-weighted imaging reveals iso- to hyperintense signals [16]. Calcification is present in approximately 50% of lesions. In addition, a ‘reverse zonal’ pattern might be observed, with heterogeneous calcification being more prominent in the center of the lesion [[17], [18]]. According to Manning et al., to diagnose extraskeletal osteosarcoma of the parotid gland, invasion by osteogenic osteosarcoma into the gland or metastasis from a primary tumor must be excluded [19]. In this case, the tumor invaded the parotid gland, but a single slice revealed attachment to the temporal bone marrow cavity. Although the criteria for extraskeletal osteosarcoma were not met, the presence of a sarcomatous tumor with central calcification could have been considered in the differential diagnosis.

The reported 5-year overall survival rate for surgically resected head and neck osteosarcoma without metastasis is 55.8% [20]. Conversely, the 5-year survival rate for surgically resected cases of nonmetastatic osteosarcoma in the extremities ranges 66%-69% [21], highlighting the poorer prognosis of head and neck osteosarcoma. Chen et al. reviewed the prognostic factors in 160 cases of head and neck osteosarcoma and identified positive surgical margins as the most significant prognostic factor for overall survival, local recurrence, and metastasis [22]. Complete resection with negative margins is essential to adequately treat head and neck osteosarcoma.

In this case, the diagnosis of osteosarcoma based on the imaging findings was difficult. Head and neck osteosarcoma is characterized by a low complete resection rate and a poor prognosis because of the complex anatomy of the region [23]. Primary osteosarcoma originating from the temporal bone is extremely rare, but preoperative imaging is crucial given the importance of complete surgical resection for prognosis. Even in the absence of overt osseous destruction, a sarcomatous mass with internal calcifications should raise suspicion for osteosarcoma. When such a lesion is encountered, radiologists should carefully assess for subtle signs of marrow infiltration.

## Ethics committee approval

This is a case report involving 1 patient; thus, institutional ethics committee approval was not required.

## Patient consent

Informed consent was obtained from the patient described in this manuscript.
